# Identification of Serum MicroRNAs as Novel Non-Invasive Biomarkers for Early Detection of Gastric Cancer

**DOI:** 10.1371/journal.pone.0033608

**Published:** 2012-03-14

**Authors:** Ming-yang Song, Kai-feng Pan, Hui-juan Su, Lian Zhang, Jun-ling Ma, Ji-you Li, Yasuhito Yuasa, Daehee Kang, Yong Sung Kim, Wei-cheng You

**Affiliations:** 1 Key Laboratory of Carcinogenesis and Translational Research (Ministry of Education), Department of Cancer Epidemiology, Peking University Cancer Hospital and Institute, Beijing, People's Republic of China; 2 Department of Pathology, Peking University Cancer Hospital and Institute, Beijing, People's Republic of China; 3 Department of Molecular Oncology, Tokyo Medical and Dental University, Tokyo, Japan; 4 Department of Preventive Medicine, Seoul National University College of Medicine, Seoul, Korea; 5 Korea Research Institute of Bioscience and Biotechnology, Medical Genomics Research Center, Daejeon, Korea; John Hopkins Medical School, United States of America

## Abstract

**Background:**

To investigate the potential of serum miRNAs as biomarkers for early detection of gastric cancer (GC), a population-based study was conducted in Linqu, a high-risk area of GC in China.

**Methodology/Principal Findings:**

All subjects were selected from two large cohort studies. Differential miRNAs were identified in serum pools of GC and control using TaqMan low density array, and validated in individual from 82 pairs of GC and control, and 46 pairs of dysplasia and control by real-time quantitative reverse transcription-polymerase chain reaction. The temporal trends of identified serum miRNA expression were further explored in a retrospective study on 58 GC patients who had at least one pre-GC diagnosis serum sample based on the long-term follow-up population. The miRNA profiling results demonstrated that 16 miRNAs were markedly upregulated in GC patients compared to controls. Further validation identified a panel of three serum miRNAs (miR-221, miR-744, and miR-376c) as potential biomarkers for GC detection, and receiver operating characteristic (ROC) curve-based risk assessment analysis revealed that this panel could distinguish GCs from controls with 82.4% sensitivity and 58.8% specificity. MiR-221 and miR-376c demonstrated significantly positive correlation with poor differentiation of GC, and miR-221 displayed higher level in dysplasia than in control. Furthermore, the retrospective study revealed an increasing trend of these three miRNA levels during GC development (*P* for trend<0.05), and this panel could classify serum samples collected up to 5 years ahead of clinical GC diagnosis with 79.3% overall accuracy.

**Conclusions/Significance:**

These data suggest that serum miR-221, miR-376c and miR-744 have strong potential as novel non-invasive biomarkers for early detection of GC.

## Introduction

Gastric cancer (GC) is the second leading cause of cancer death in the world, with nearly half occurring in China [1], [2]. The prognosis of GC varies remarkably by the stage of cancer with the 5-year relative survival rate reaching 90% in Stage I but less than 5% in Stage IV [3]. Thus, early detection of GC is a key measure to reduce the mortality and improve the prognosis of GC.

Although gastroscopic screening for GC is highly reliable, it is invasive and costly, particularly for the developing countries. Therefore, much effort has been made to develop less expensive preliminary screening tests in easily accessible specimens. However, many previously investigated analytes, such as pepsinogen (PG) I/II ratio, carcinoembryonic antigen (CEA) and carbohydrate antigen 19-9 (CA 19-9), were not sensitive and specific enough for GC screening [3], [4]. Thus, there is an urgent need for new non-invasive biomarkers to improve the early detection of GC.

MicroRNAs (miRNAs) are an abundant class of small non-coding RNAs that negatively regulate gene expression by base pairing with the 3′-untranslated region of target mRNAs, resulting in either mRNA cleavage or translational repression [5], [6]. Increasing evidence has shown that miRNAs are involved in various biological processes, including development, cell differentiation, proliferation and apoptosis [7], and participate in human carcinogenesis as oncogenes or tumor suppressors [8]. Studies have indicated that miRNA expression profile varies among tumor types and can differentiate cancer and normal tissues [9], [10]. Recently, circulating miRNAs have been suggested great potential as biomarkers for many cancers, including GC [11]–[16]. However, the value of circulating miRNAs in early detection of GC has not been reported yet.

Since 1989, we have conducted a series of studies in Linqu County, a high-risk area of GC in Shandong Province, China, including a population-based cohort study of precancerous gastric lesions [17], [18], and a randomized trial to inhibit the progression of gastric lesions [19]. This cohort allows us to investigate the dynamic changes in circulating miRNA levels during GC development, and provides us a unique opportunity to explore the potential of circulating miRNAs in early detection of GC.

Herein, we report the results of differential miRNAs in the serum of GC and dysplasia (DYS), and a retrospective study designed to evaluate the dynamic changes during GC development.

## Materials and Methods

### Study design and population

The details of study population, procedures of endoscopic examination, criteria of gastric histopathology and follow-up have been described elsewhere [17]–[19]. Briefly, an endoscopic screening survey was launched in 1989 among 3399 residents aged 35–64 years in Linqu County, a high-risk area of GC, and the subjects without GC diagnosis were subsequently followed up with the repeated endoscopic examination conducted in 1994 [17], [18]. In 1995, a randomized, placebo-controlled intervention trial was initiated in this population until 2003, when repeated endoscopic examination was performed [19]. All subjects underwent blood draw in both the baseline and end-point surveys of each study, and some with advanced gastric lesions, such as intestinal metaplasia (IM) or DYS, also provided blood and received endoscopic examination in 1992 and 1999.

**Figure 1:**
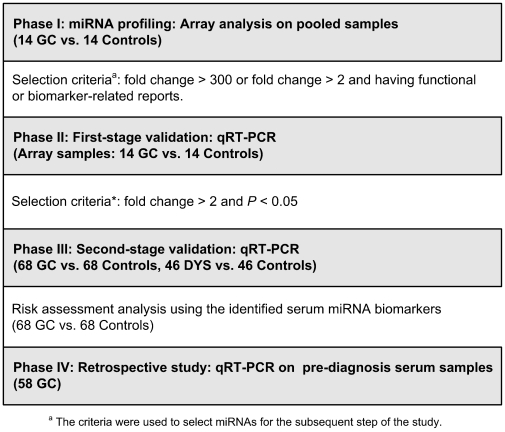
The flow chart of the study design.

In the current study, a multi-stage, nested case-control study from two large cohorts was designed to identify and validate the differential serum miRNAs in GC and DYS, and a retrospective study was conducted to investigate the potential of candidate serum miRNAs in early detection of GC (Figure 1).

This study was divided into four stepwise phases. In phase I, serum pools from 14 GC patients and 14 age- and sex-matched controls with the diagnosis of superficial gastritis (SG) or mild chronic atrophic gastritis (CAG) were used to generate miRNA profiles by array analysis. Differential miRNAs were identified, and the extremely upregulated miRNAs or moderately upregulated miRNAs having functional or biomarker-related reports were selected for further analysis. In phase II, identified miRNAs were validated using quantitative reverse transcription-polymerase chain reaction (qRT-PCR) on the individual serum samples of 14 GCs and controls that were used for the array test in phase I. In phase III, the significantly altered miRNAs were subsequently validated in 68 GC and 46 DYS patients as well as their age- and sex-matched controls. Receiver operating characteristic (ROC) curve-based risk assessment analysis was then performed among the 68 GC-control pairs to evaluate the discriminating effect of serum miRNAs on GC. Each subject was assigned into either high- or low-risk groups by comparing the expression levels of miRNA biomarkers to the corresponding cut-off values derived from ROC curve. In phase IV, a retrospective study was conducted in 58 GC patients, who provided at least one eligible pre-diagnosis serum sample in the long-term follow-up study as described above, to explore the temporal trend of potential miRNA biomarkers and determine their value in early GC detection. The whole study was approved by the Institutional Review Board of Peking University Cancer Hospital & Institute, and all subjects gave written informed consent.

### Sample processing and RNA extraction

Up to 5 ml of whole blood from each fasting participant was collected with cases and controls done at the same year. Blood samples were allowed to stand for 30–40 min and serum separation was accomplished by centrifugation at 965 g for 15 min. The supernatant serum was recovered and stored at −80°C until analysis. RNA was isolated from 100 µL serum using the miRNeasy Mini Kit (Qiagen, Germany) following the manufacturer's protocol with minor modification [20]. For miRNA profiling, two pools were created by combining the mixtures of upper aqueous phase after phase separation and ethanol from 14 GC patients (12 male, 2 female; age, 62 (SD 6.4) years) and 14 sex- and age-matched controls (age, 61 (SD 5.9) years), respectively. After binding to the membrane of RNeasy Mini spin column and subsequent washing, RNA was eluted with 40 µL of RNase-free water. For all qRT-PCR experiments, RNA extracted from 100 µl of serum was eluted with 100 µL of RNase-free water. To normalize the sample-to-sample variation in RNA isolation step, synthetic ath-miR-159a was added to each serum sample for miRNA profiling while cel-miR-39 was added for qRT-PCR analysis as described by Mitchell *et al* [11].

### MiRNA profiling

MiRNA profiling was performed using TaqMan low density array A (v2.0) according to the manufacturer's recommended protocol (Applied Biosystems, Foster City, CA, USA). This qRT-PCR profiling platform consists of the one 384-well microfluidic card in which 377 miRNAs can be analysed together with three endogenous controls (U6, RNU44 and RNU48) and one negative control unrelated to human (ath-miR-159a). In brief, the RNA pooled separately from 14 GC patients and matched controls as described above was reverse transcribed using the TaqMan miRNA reverse transcription kit and TaqMan miRNA multiplex RT assays (human pool; Applied Biosystems). For each RNA pool, 3 µl of total RNA was added to each of the multiplex reverse transcription reactions.

To increase the amount of cDNA available for analysis, enrichment of target genes was performed with a preamplification step. The 25-µl reaction mixture consisted of 2.5 µl of undiluted cDNA combined with 12.5 µl of TaqMan PreAmp Master Mix (2×), 2.5 µl of Megaplex PreAmp Primers (10×), and 7.5 µl of nuclease-free water. The preamplification step was performed on a GeneAmp PCR System 9700 (Applied Biosystems) following the manufacturer's protocol.

For real-time PCR analysis, the product was diluted by adding 75 µl of nuclease-free water, and 9 µl of the diluted mixture was subsequently combined with 450 µl of TaqMan 2×Universal PCR Master Mix without uracil-N-glycosylase and 441 µl of nuclease-free water. After loading 100 µl of each multiplex pool mixture, the array was centrifuged and sealed. Amplification was performed on an Applied Biosystems 7900 HT thermal cycler (Applied Biosystems) using the manufacturer's recommended cycling conditions. Data were analysed using RQ Manager Software version 1.2 and DataAssist™ Software version 2.0 (Applied Biosystems). After normalization using the spiked-in ath-miR-159a, the fold change of miRNAs levels in GC compared to control was calculated by the 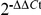 method.

### MiRNA quantification by real-time qRT-PCR

Reverse transcription was performed using the TaqMan miRNA reverse transcription kit and miRNA-specific stem-loop primers (Applied Biosystems). The 7.5-µl reaction mixture consisted of 0.75 µl of 10×Reverse-transcription buffer, 0.095 µl of RNase inhibitor (20 units/µl), 0.075 µl of 100 mM dNTPs with dTTP, 0.5 µl of MultiScribe reverse-transcriptase (50 units/µl), 1.5 µl of primer, 2.5 µl of RNA, and 2.08 µl RNase-free water. Reverse transcription was performed on a T professional PCR System (Biometra, Germany) following the manufacturer's protocol.

Real-time PCR was performed in duplicate and included no-template negative controls. For the 20-µl reaction, cDNA (3 µl) was combined with 10 µl of TaqMan 2×Universal PCR Master Mix without uracil-N-glycosylase, 1 µl of the TaqMan miRNA assay mix, and 6 µl of water. Amplification was performed on a 7500 Real-Time PCR system (Applied Biosystems) following the manufacturer's protocol. The expression levels of miRNAs were normalized to the spiked-in cel-miR-39, and were calculated using the 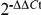 method.

### Statistical analysis

The paired *t* test was used to examine the difference in mean age between groups. The Wilcoxon test or Mann–Whitney U test was used to compare the serum miRNA levels of different groups where appropriate. ROC curves were established to evaluate the diagnostic effects of miRNAs, and the generalized linear model with repeated measures was employed to analyse the temporal trends of serum miRNA levels during GC development after logarithmic transformation. All *P* values were two-sided and less than 0.05 was considered statistically significant. All statistical analyses were performed using the Statistical Package for the Social Sciences (SPSS 13.0, Chicago, IL).

## Results

### Subject characteristics

A total of 82 GC patients, 46 subjects with DYS, and 128 controls with SG or mild CAG were included in this study. As shown in Table 1, there was no significant difference in ages between patients with GC or DYS and their corresponding controls (*P* = 0.329 and 0.214, respectively).

**Table 1:** Subject information of the case-control study.

Variables	GC n = 82	Control n = 82	DYS n = 46	Control n = 46
Age (year, Mean ± SD)	60.4±7.5	60.3±7.5	58.3±10.4	58.1±10.0
Gender (%)				
Male	58 (70.7)	58 (70.7)	39 (84.8)	39 (84.8)
Female	24 (29.3)	24 (29.3)	7 (15.2)	7 (15.2)
Lauren's types (%)				
Intestinal	32 (39.0)			
Diffuse	6 (7.3)			
Mixed	1 (1.2)			
Uncertain	1 (1.2)			
Missing	42 (51.3)			
Histology (%)				
Adenocarcinoma	71 (86.6)			
Signet ring cell	3 (3.7)			
Squamous carcinoma	1 (1.2)			
Missing	7 (8.5)			
Differentiation (%)				
Well	5 (6.1)			
Moderately	19 (23.2)			
Poorly	43 (52.4)			
Missing	15 (18.3)			
Metastasis (%)				
Yes	22 (26.8)			
No	31 (37.8)			
Missing	29 (35.4)			

### MiRNA profiling

The differentially expressed miRNAs were identified in serum pools of 14 GCs and 14 controls using TaqMan low density array. Among 377 miRNAs analysed, 99 showed >2-fold upregulated and 40 showed <0.5-fold downregulated changes in GC serum pool (Table S1). Sixteen upregulated miRNAs (miR-221, miR-744, miR-376c, miR-191, miR-27a, let-7e, miR-27b, miR-222, miR-21, miR-18a, miR-19a, miR-17, miR-106b, miR-106a, miR-20a, and miR-155) demonstrating remarkable expression changes in GC serum and having functional or differential expression reports in GC tissue were selected as the candidates for the fist-stage validation [21]–[28].

### First-stage validation

The expressions of 16 candidate miRNAs were measured in individual serum samples of 14 GCs and controls that were used for array test. Consistently with the profiling results, all 16 miRNAs showed higher serum levels in GC group than in control group (Figure S1). Among them, 9 serum miRNAs (miR-221, miR-744, miR-376c, miR-191, miR-27a, let-7e, miR-27b, miR-222, and miR-21) had >2 average fold change in GC group compared to control group (*P*<0.05), and were thus chosen for further validation.

### Second-stage validation

**Figure 2:**
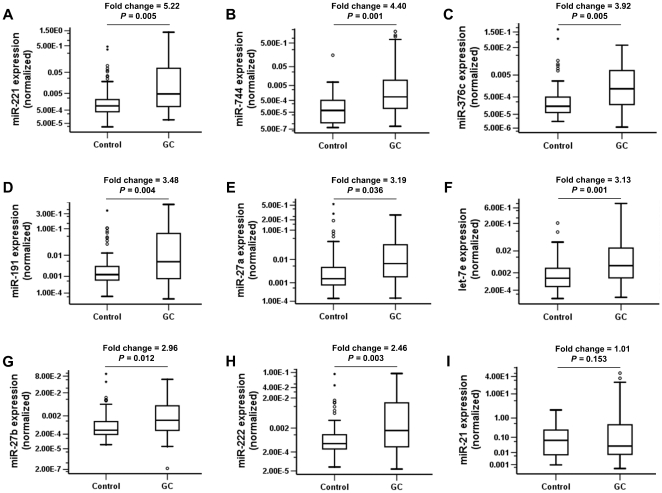
Serum levels of the selected miRNAs in 68 pairs of GC and control subjects in the second-stage validation. The median fold changes of miRNA levels comparing GC with control were given and the Wilcoxon tests were performed to examine the difference between two groups. The relative levels of miRNAs (log10 scale at Y-axis) were normalized to the spiked-in cel-miR-39.

We examined the 9 miRNA expressions on a large set of serum from 68 pairs of GC and control. As shown in Figure 2, 8 out of the 9 miRNAs (miR-221, miR-744, miR-376c, miR-191, miR-27a, let-7e, miR-27b, and miR-222) demonstrated significantly elevated levels in GC group with more than 2-fold change (*P*<0.05), and these miRNAs were selected for the following analyses.

### Risk assessment of GC cases using a panel of miRNAs

**Figure 3:**
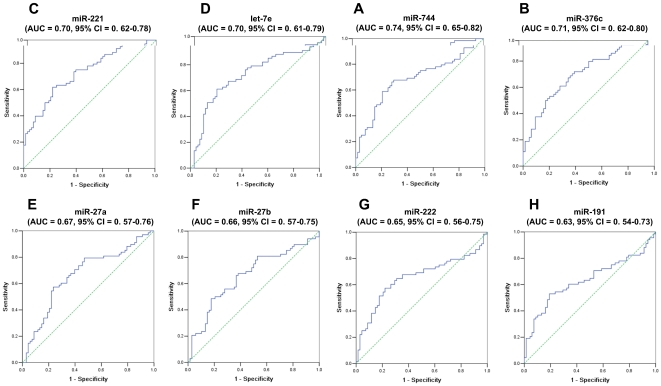
Receiver operating characteristics (ROC) curve analysis using eight serum miRNAs for discriminating 68 GCs from 68 controls. Serum (A) miR-744, (B) miR-376c, (C) miR-221 and (D) let-7e yielded the largest areas under the ROC curves (AUCs).

To evaluate the discriminating effect of serum miRNAs on GC, ROC curves were established for each of the 8 miRNAs using 68 pairs of GC and control in the second-stage validation (Figure 3). The results revealed that miR-744, miR-376c, miR-221 and let-7e yielded the largest areas under the ROC curves (AUCs) and might have the potential as biomarkers for GC detection.

Risk assessment based on the four miRNAs was used to distinguish serum samples of GC cases from controls. For each miRNA, we firstly calculated the optimal cut-off value of the relative expression level (miR-744: 5.49×10^−4^; miR-376c: 6.05×10^−4^; miR-221: 1.47×10^−3^; let-7e: 1.61×10^−3^), at which the Youden's index (sensitivity+specificity-1) for GC diagnosis was largest at the ROC curve. Then we divided the subject into a high-risk group, representing the possible GC case, when the serum level of any of the four miRNAs was equal or greater than the corresponding cut-off value, and a low-risk group, representing the control, when the serum levels of all the four miRNAs were less than the cut-off values.

According to this assessment criterion, 56 GCs and 32 controls were correctly predicted as GC and non-GC cases, producing the sensitivity of 82.4% and the specificity of 47.1%. Since let-7e demonstrated the smallest value and widest confidence interval of AUC among the four miRNAs in the ROC curve analysis (Figure 3), we repeated the above risk assessment after excluding let-7e and obtained a higher specificity (40/68, 58.8%) while the sensitivity remained unchanged, indicating that the contribution of let-7e to differentiating GCs from controls was negligible, and the three-miRNA panel (miR-221, miR-376c and miR-744) was a more efficient combination of biomarkers for GC diagnosis.

To further examine the potential of this panel for early detection of GC, we assessed a subgroup of 30 GC patients in early stage, and 22 were correctly predicted as GC cases with the sensitivity of 73.3%.

### Association between serum miRNA expression and differentiation grades of GC

**Figure 4:**
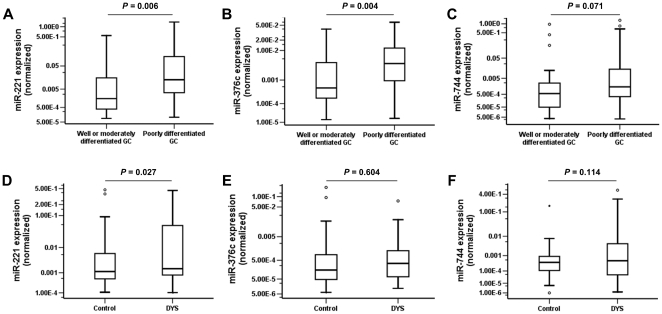
Association of the selected serum miRNA levels with GC differentiation grades and DYS. (A–C) Box plots of serum miR-221, miR-376c and miR-744 levels in 24 well or moderately differentiated and 43 poorly differentiated GC patients. (D–F) Box plots of serum miR-221, miR-376c and miR-744 levels in 46 DYS subjects and matched controls. The Mann-Whitney U tests (A–C) and the Wilcoxon tests (D–F) were performed to examine the difference between two groups, respectively. The relative levels of miRNAs (log10 scale at Y-axis) were normalized to the spiked-in cel-miR-39 (A–F).

Furthermore, we sought to investigate the correlation between expression levels of miR-221, miR-744 and miR-376c, and differentiation grades of GC. As shown in Figure 4A–C, all the three miRNAs showed higher serum levels in poorly differentiated GC group (43 GCs including 29 male and 14 female; age, 60 (SD 8.3) years) than in well or moderately differentiated GC group (24 GCs including 19 male and 5 female; age, 60 (SD 6.2) years). Among them, serum miR-221 and miR-376c demonstrated significantly positive correlation with poor differentiation of GC (*P* = 0.006 and 0.004, respectively).

### Expression levels of serum miRNA biomarkers in subjects with DYS

To further elucidate the relevance of these three miRNAs with premalignant gastric lesion, we examined their expressions in 46 pairs of DYS and control. As shown in Figure 4D–F, a significantly elevated level of miR-221 was found in DYS group when compared to controls (*P* = 0.027), while no significant difference was found for miR-376c or miR-744. Further risk assessment analysis following previously established procedures revealed that this three-miRNA panel could distinguish DYS from control with the sensitivity of 56.5% and the specificity of 47.8%.

### Dynamic changes of serum miRNA biomarkers during GC development

In the retrospective study, the dynamic changes of the three serum miRNA biomarkers were detected on 58 GC patients who were divided into four groups according to the time interval from blood draw to GC diagnosis: pre-diagnosis 2–5-year, 6–9-year, 10–14-year and ≥15-year groups (Table 2). Some subjects may simultaneously exist in two or more than two groups since they provided serum samples in multiple time points of the follow-up period.

**Table 2:** Information of the 58 GC cases for the retrospective study.

Variables	Pre-diagnosis ≥15-year group n = 12	Pre-diagnosis 10–14-year group n = 36	Pre-diagnosis 6–9-year group n = 27	Pre-diagnosis 2–5-year group n = 29
Age (year, Mean ± SD)	54.3±4.9	55.2±10.3	58.0±9.6	60.2±8.4
Gender (%)				
Male	5 (41.7)	25 (69.4)	20 (74.1)	20 (69.0)
Female	7 (58.3)	11 (30.6)	7 (25.9)	9 (31.0)
Pathologic diagnosis (%)				
Superficial gastritis	-	-	3 (11.1)	-
Chronic atrophic gastritis	1 (8.3)	4 (11.1)	2 (7.4)	-
Intestinal metaplasia	4 (33.3)	19 (52.8)	9 (33.3)	10 (34.5)
Dysplasia	5 (41.7)	12 (33.3)	12 (44.4)	19 (65.5)
Missing	2 (16.7)	1 (2.8)	1 (3.8)	-

**Figure 5:**
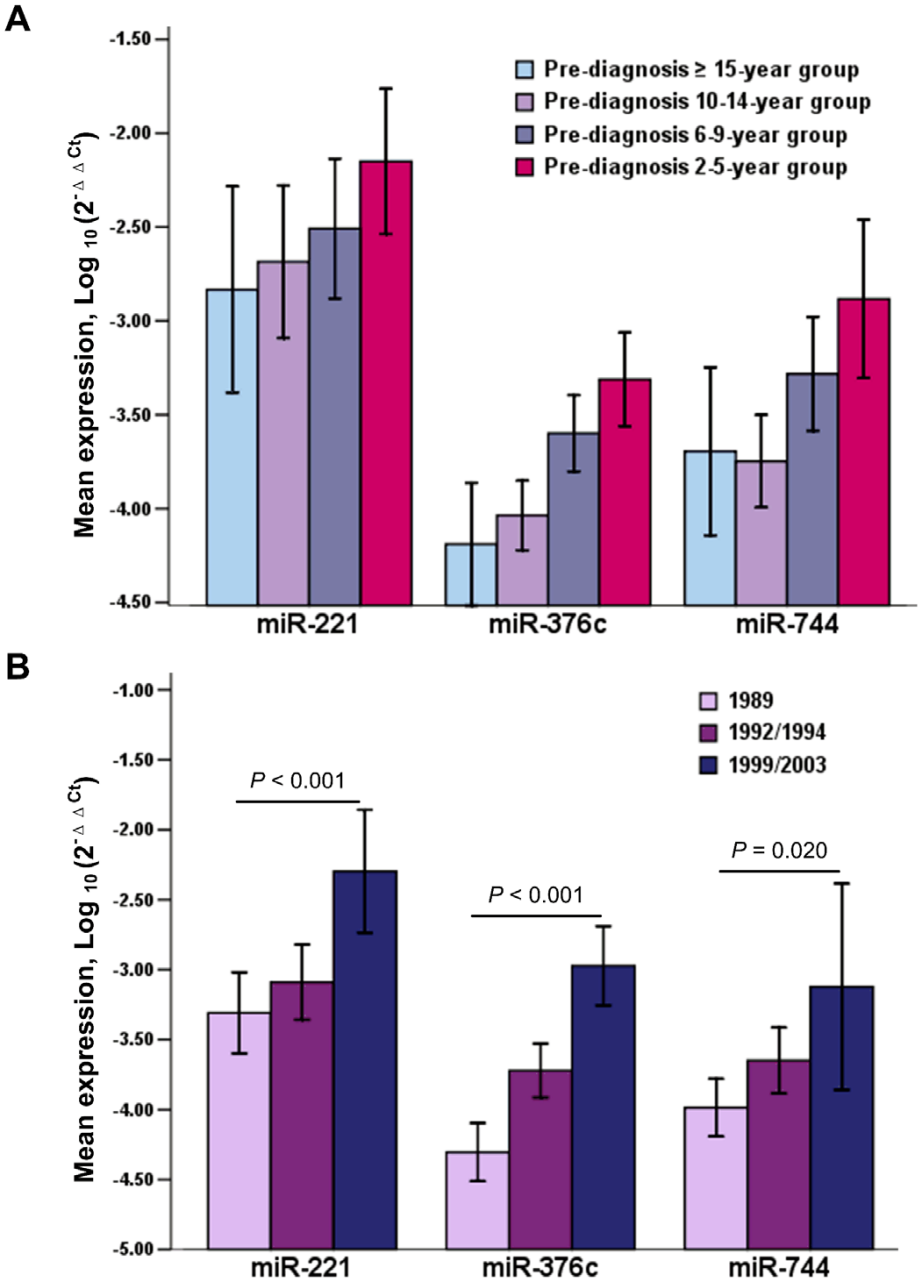
The temporal trends of serum miR-221, miR-376c and miR-744 levels during GC development. (A) In the retrospective study, miRNA levels were examined in the pre-diagnosis serum samples of 58 cases that were classified into four groups according to the time interval between blood collection and GC diagnosis. (B) The trend tests of miRNA levels over time were performed in 20 GC cases who provided three serum samples at three time points of the follow-up (in the years of 1989, 1992 and 1999; or in the years of 1989, 1994 and 2003). The mean values of relative miRNA levels that were normalized to the spiked-in cel-miR-39 and transformed on a logarithmic scale were shown with the error bar representing 95% CI (A–B).

As shown in Figure 5A, all three serum miRNAs displayed markedly increasing expression during GC development, except slight increase of serum miR-744 in the pre-diagnosis ≥15-year group. Further trend test showed a statistically significant linear trend in the three miRNA levels among 20 GC patients who contributed three serum samples at different time points during the follow-up period (Figure 5B).

Finally, we evaluated the potential of miRNA biomarkers for early prediction of GC in 29 patients, who had provided serum samples at 2–5 years before GC diagnosis. According to the risk assessment criterion established before, 23 of the 29 GC patients (79.3%) were correctly classified as the represented GC cases by this three-serum miRNA panel.

## Discussion

The aim of this study was to identify differential miRNAs in the serum of GC and DYS, and investigate the potential of serum miRNAs as biomarker for early detection of GC. We found the significantly elevated levels of serum miR-221, miR-376c and miR-744 in GC patients through systematic microarray-based screening and two-stage validation. This three-serum miRNA panel could distinguish GCs from controls and showed an increasing trend during GC development. To the best of our knowledge, this is the first population-based study in exploring the dynamic changes of serum miRNAs associated with GC and its precursors in a high-risk area of GC.

The incidence of GC varies widely throughout the world with the highest rates occurring in eastern Asia, including Japan, Korea and China [1], [2]. In China, most of GCs are diagnosed at advanced stages resulting in poor prognosis with an average 5-year survival rate <30%, while in Japan and Korea over 60% of GCs are diagnosed at early stage by community-based endoscopic screening with 5-year survival rate >80% [29], [30]. Although the impact of early detection of GC by endoscopic screening is significant in Linqu, there has been only a few population-based screening for GC in China due to high cost and lack of skilled endoscopists.

GC is an end result of multi-stage gastric lesions with different biological features, which provides an opportunity to detect GC in early stage by using biomarkers. In our previous study, we found the ratio of serum PG I/II monotonically declined with the severity of gastric lesions, however, the sensitivity and specificity for predicting advanced gastric lesions and GC were poor [31]. Recently, accumulating evidence indicated that miRNAs play important roles in oncogenesis, and tumor-derived miRNAs can enter the circulation in a remarkably stable form [11], [32], [33], suggesting that miRNAs could be used as novel non-invasive biomarkers for early detection of GC.

More recently, a study identified four upregulated GC-associated miRNAs (miR-17-5p, miR-21, miR-106a and miR-106b) [34], which also displayed higher expression levels in GC serum on our array, whereas the striking downregulation of let-7a in their study was not observed by us. Another study found five elevated serum miRNAs (miR-1, miR-20a, miR-27a, miR-34 and miR-423-5p) in GC patients [13], whereas only miR-20a and miR-27a were upregulated on our array, and miR-27a was validated to be remarkably overexpressed in GC patients. The partially inconsistent results may reflect the differences in sample types, screening tools or quantification methods [35].

Among the three serum miRNAs identified in our study, miR-221 has been found abnormally upregulated in tissues of many cancer types, such as gastric [24], colorectal [21], prostate [36] and breast cancer [37]. MiR-221 could post-transcriptionally suppress p27 and p57, thus facilitating G1/S phase transition and enhancing cell proliferation and tumor growth [24], [36], [38], [39]. Studies have shown that the expression level of miR-221 in plasma was associated with overall survival of colorectal cancer [40] and the progression of prostate cancer [41]. Only a few studies have explored the functional relevance of miR-376c, such as miR-376c could enhance proliferation, survival and chemoresistance of ovarian cancer cell by targeting ALK7 [42]. For miR-744, its expression pattern, physiological function and relationship with carcinogenesis are unknown at present.

In our current study, we found this three-miRNA panel could distinguish serum samples of GC from controls with a sensitivity of 82.4% and specificity of 58.8%. In addition, our study indicated that among 30 early-stage GC patients, 22 (73.3%) were correctly classified, suggesting this panel might serve as a biomarker for early detection of GC. It also raises the possibility that this panel of miRNAs may be useful as a preliminary screening method for GC in general population at high risk of GC.

We were also interested in exploring the associations between these three serum miRNAs and differentiation grades of GC. By comparing miRNA levels in serum of GC patients with various grades, we found a higher serum miRNA expression in poorly differentiated GC. This result revealed that high expression level of these serum miRNAs in GC patients was associated with the advanced stage of GC. Although the mechanism of this phenomenon is unclear, the high level of these miRNAs in serum may have some value for indicating the progression and treatment selection of GC.

We further assessed whether this three-miRNA panel could predict advanced gastric lesions, such as DYS. We found only miR-221 displayed an elevated expression level in DYS, suggesting this panel was more specific for GC. Despite the limited effect of this panel on DYS detection, miR-221, one of the three serum miRNAs, may be useful for identifying those DYS subjects at the extremely high risk of developing GC. Interestingly, by prospectively tracking the evolution of these DYS subjects, we found the lower rate of regression to the less severe lesions and one incident GC in the predicted high-risk group who had higher level of serum miR-221 (data not shown).

By retrospectively examining the expression of three serum miRNAs in pre-diagnosis samples of GC patients, we found an increasing trend during GC development, and further analysis indicated that this panel could predict GC up to 5 years ahead of clinical GC diagnosis. These results strongly supported the value of these three miRNAs for predicting GC occurrence. Combining this three-serum miRNA signature with pathological diagnosis may enhance early detection of GC in subjects with advanced gastric lesions, such as IM and DYS.

To rule out the possibility that increased age and shorter storage period of serum samples contributed to higher level of miRNAs over time, we analysed miRNA expression in 82 controls who underwent blood draw at the same time points with GC patients. No significant difference was observed during the follow-up period (data not shown), suggesting that the rising serum miRNA levels throughout GC development could not be attributed to increased age and supporting that miRNAs were present in serum in a stable form. Accordingly, circulating miRNAs might be used as a biomarker to develop a non-invasive test for GC detection in the future because of its easy accessibility, high stability and low assay cost.

Our study has several strengths. Firstly, all subjects came from a high-risk population of GC and underwent rigorous pathologic diagnosis, which justified our hypothesis that the observed difference in serum miRNA levels between GCs and controls could be attributed mostly, if not all, to the neoplastic transformation. Secondly, our population-based study with a high compliance and more than 20 years' follow-up enhanced the reliability of the study, and provided the first evidence on dynamic changes of serum miRNAs during GC development as well as their potential as biomarkers to identify high-risk individuals of GC.

This study also has some limitations. Because very few subjects were diagnosed with normal gastric mucosa in this population [17], we used subjects with SG/mild CAG as controls. However, compared to studies using generally normal subjects as control, our pathologic diagnosis-based control selection strategy diluted the disparity between cases and controls, and thus could only cause the underestimation of results. In addition, the findings of this preliminary study with small sample size necessitate further confirmation in large prospective studies and the functional relevance of the newly identified miRNAs needs to be further clarified.

In conclusion, through this population-based study we identified a set of differential serum miRNAs (miR-221, miR-376c and miR-744) in GC and proposed their potential as novel non-invasive biomarkers for early detection of GC.

## Supporting Information

Figure S1 **Serum levels of the 16 selected miRNAs in 14 pairs of GC and control subjects in the first-stage validation.** The median fold changes of miRNA levels comparing GC with control were given and the Wilcoxon tests were performed to examine the difference between two groups. The relative levels of miRNAs (log10 scale at Y-axis) were normalized to the spiked-in cel-miR-39.
(JPG)

Table S1 (XLS)
