# Supplementary figures and images for: Identification of Serum MicroRNAs as Novel Non-Invasive Biomarkers for Early Detection of Gastric Cancer
Source: PLoS One. 2012 Mar 14;7(3):e33608. doi: 10.1371/journal.pone.0033608 (PMC3303856; doi:10.1371/journal.pone.0033608)

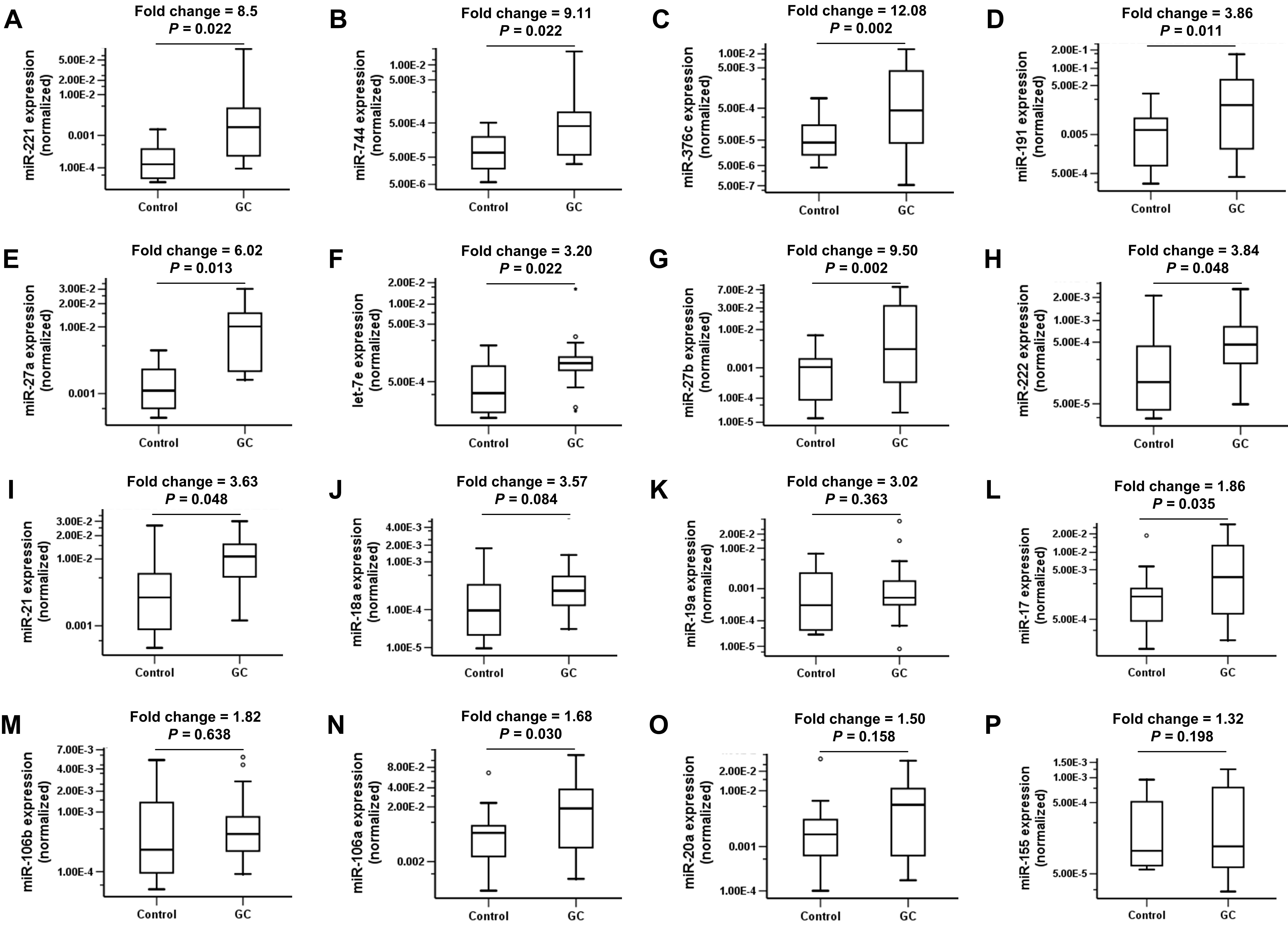

Supplement: Figure S1 — Serum levels of the 16 selected miRNAs in 14 pairs of GC and control subjects in the first-stage validation. The median fold changes of miRNA levels comparing GC with control were given and the Wilcoxon tests were performed to examine the difference between two groups. The relative levels of miRNAs (log10 scale at Y-axis) were normalized to the spiked-in cel-miR-39. (JPG) [file pone.0033608.s001.JPG]
